# Maintenance and Decline of Neuronal Lysosomal Function in Aging

**DOI:** 10.3390/cells14241976

**Published:** 2025-12-12

**Authors:** Ruiling Zhong, Claire E. Richardson

**Affiliations:** Department of Genetics, University of Wisconsin-Madison, Madison, WI 53706, USA

**Keywords:** lysosome, neuron, aging, endolysosome, autolysosome, autophagy, TFEB

## Abstract

Lysosomes are central effectors of cellular maintenance, integrating the degradation of damaged organelles and protein aggregates with macromolecule recycling and metabolic signaling. In neurons, lysosomes are particularly crucial due to the cells’ long lifespan, polarized architecture, and high metabolic demands. Proper regulation of lysosomal function is essential to sustain proteostasis, membrane turnover, and synaptic integrity. Although lysosomal dysfunction has been extensively studied in neurodegenerative diseases, far less is known about how lysosomal capacity and function are maintained—or fail to be maintained—with age in non-diseased neurons. In this review, we summarize current understanding of neuronal lysosomal dynamics, discuss methodological challenges in assessing lysosomal capacity and function, and highlight recent advances that reveal age-associated decline in neuronal lysosomal competence.

## 1. Introduction

Lysosomes are membrane-bound degradative and signaling organelles essential for maintaining cellular homeostasis [1,2]. They contain an array of hydrolases within an acidic lumen that break down proteins, lipids, and nucleic acids delivered through the endocytic, autophagic, and phagocytic pathways. The resulting metabolites are released into the cytoplasm and recycled into biosynthetic and energetic pathways, linking lysosomal degradation to cellular metabolism and renewal. Beyond these canonical roles, lysosomes also act as organizing centers for metabolic signaling and stress response pathways such as mTORC1, AMPK, and calcium signaling [3,4]. Within individual cells, the balance between cellular growth and catabolism fluctuates in response to environmental and physiological conditions, necessitating the adjustment of lysosomal degradative capacity to meet these dynamic demands [1,5]. Because lysosomes continuously fuse with endosomal and autophagic compartments and then reform from endolysosomes and autolysosomes, a suite of lysosomes and related compartments of heterogeneous size, luminal pH, and molecular composition together define a cell’s lysosomal capacity [6,7,8]. For clarity, we use “lysosomal capacity” to mean the product of (A) the abundance of competent degradative lysosomal compartments and (B) their per-organelle degradative potential; “lysosomal function” refers to operational readouts including luminal pH, hydrolase activity, fusion/fission competence, cargo flux, and trafficking.

In neurons, proper lysosomal function is both especially important and unusually challenging. It is important because most neurons are generated during development and maintained throughout the lifespan of the animal in which they reside. This necessitates robust proteostasis and organelle quality control, including degradation and replacement. The exceptional morphological complexity of neurons contributes to the challenge of generating adequate lysosomal function: degradative lysosomes predominantly reside in the soma, but lysosomal degradative cargo resides throughout the neuron, including the distal axons and dendrites [9,10]. This necessitates the sustained transport of endosomes, autophagosomes, and other lysosomal compartments to the neuron cell body. Likely due to a combination of these circumstances, neurons are particularly sensitive to impairments in lysosomal function [9,11].

Much of the research on neuronal lysosomes has centered around the study of neurodegenerative diseases [12,13,14]. In Alzheimer’s disease (AD), Parkinson’s disease (PD), and other age-associated neurodegenerative diseases, lysosomal and autophagic defects contribute to the buildup of toxic proteins and organelle damage [15]. These pathological contexts highlight the critical role of lysosomal function in neuronal maintenance. Further, lysosomal decline is not limited to disease states. It has long been appreciated that neuronal lysosomal structure and function change in aging [16]. Age-associated neuronal lysosomal phenotypes include increased size and heterogeneity, altered positioning within neurites, and accumulation of undegraded substrates such as lipofuscin [17]. These changes in neurons parallel age-associated lysosomal alterations in a range of other tissue types from *C. elegans* to humans [18,19,20,21]. As described below, recent research building on the momentum of interest and methodological improvements has made it increasingly clear that lysosomal decline is a physiological feature of neuronal aging even in the absence of disease.

How neuronal lysosomal capacity is maintained across the lifespan and what mechanisms drive neuronal lysosomal decline are important questions of ongoing research. Recent work highlights the roles of transcriptional regulators, including Transcription Factor EB (TFEB), signaling pathways that tune lysosomal activity to metabolic state, regulation of lysosomal compartment trafficking, and mechanisms to sense and ameliorate lysosomal stress [1,22,23,24,25].

In this review, we synthesize current knowledge of neuronal lysosomal functions, the evidence for their decline during aging, and the transcriptional and signaling mechanisms that regulate their capacity. We feature emerging insights from experimental organisms and recent methodological advances that allow neuronal lysosomal function to be visualized and quantified.

## 2. Lysosomal Functions in Neurons

### 2.1. Catabolism and Recycling

Degradative lysosomes are the terminal organelles for a wide range of substrates, including proteins, lipids and organelles [26]. Their degradative capacity, which is generated by both the abundance of lysosomes and their functionality, is particularly critical in neurons, where turnover of neuronal proteins, organelles, and other macromolecules sustains functionality across an animal’s lifespan.

**Figure 1:**
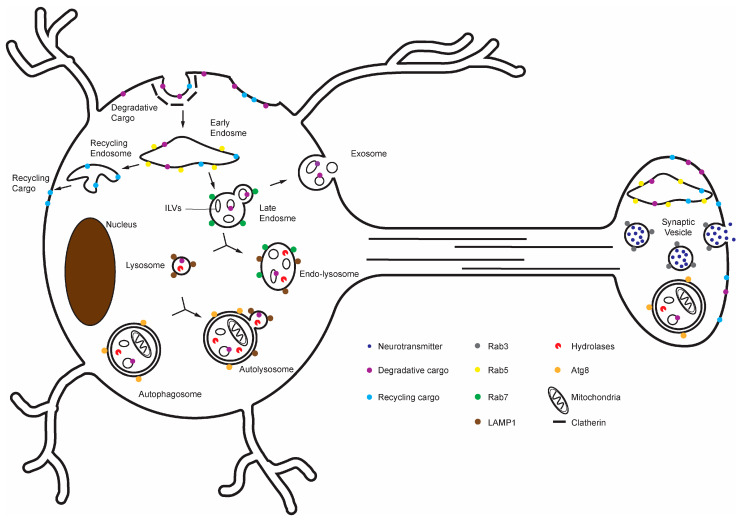
Overview of the delivery of lysosomal degradative cargoes via endo-lysosomal and autophagic pathways in the neuron. Surveillance and sorting of lysosomal degradative cargoes occur throughout the neuron, but degradative lysosomes predominantly reside in the soma.

One major route of cargo delivery to lysosomes is the endo-lysosomal pathway (Figure 1). In addition to endocytosis, cytosolic proteins can enter this pathway through microautophagy, in which late endosomes and lysosomes directly engulf cytosolic material [27]. Following endocytosis, early endosomes function as sorting stations that direct cargo either toward degradation or back to the plasma membrane [28,29,30]. Recycling cargo can be returned to the cell via retromer-mediated retrieval subdomains or by bulk membrane flow [31,32,33]. By contrast, cargo destined for degradation is recognized by the Endosomal Sorting Complexes Required for Transport (ESCRT) machinery, which binds ubiquitinated transmembrane proteins and sorts them into intraluminal vesicles (ILVs) within maturing endosomes, generating multivesicular bodies (MVBs) [32,34,35]. Some ILVs are released to the extracellular space as exosomes [36,37,38], whereas others remain within the organelle as it acidifies and transitions from a RAB5-positive early endosome to a RAB7-positive late endosome [39]. Late endosomes subsequently fuse with pre-existing lysosomes to form acidic, cathepsin-active endolysosomes, a process mediated by SNARE proteins including VAMP7 [40,41]. For example, during synaptic vesicle cycling, synaptic vesicle proteins retrieved from the plasma membrane often enter early endosomes, where most are recycled back into synaptic vesicles, but a subset is trafficked into late endosomes and delivered to lysosomes for degradation [42,43,44].

Autophagy provides several parallel routes for cargo degradation. In macroautophagy, autophagosomes form throughout dendrites and presynaptic axonal regions, where they engulf cytoplasmic material [45]. These autophagosomes fuse with lysosomal compartments in the neurites, which promotes retrograde trafficking, and in the soma to complete degradation [7,46,47,48,49]. Macroautophagy is particularly important in neurons for removing damaged mitochondria (mitophagy) and protein aggregates that cannot be refolded (aggrephagy) [45,50,51,52,53]. Fusion of autophagosomes with lysosomes is mediated by the BORC complex, the HOPS complex, and the autophagosomal SNARE syntaxin 17, whereas lysosomal trafficking regulator (LYST) facilitates lysosome reformation from autolysosomes [54,55,56]. Defects in autophagosome–lysosome fusion lead to the accumulation of autophagosomes, a phenotype commonly observed in neurodegenerative disease models [57,58]. The term macroautophagy encompasses the entire process from autophagosome initiation through autolysosomal degradation and lysosome reformation. Dysfunction of macroautophagy is considered a hallmark of aging, meaning that its occurrence correlates with age, and it drives functional decline in aging [59,60,61,62]. Different studies pinpoint impairments at distinct stages of macroautophagy in aging—some upstream of lysosomes, others that involve lysosomal steps. This distinction has implications for therapeutic strategies and is considered below. Chaperone-Mediated Autophagy (CMA) is another route of lysosome-mediated protein degradation, and it involves the translocation of degradative cargo proteins across the lysosomal membrane [63,64].

In addition to continuous fusion and reformation with endosomes and autophagosomes, lysosomes undergo fission, which adjusts lysosomal compartment number, size and positioning [65]. Spatacsin and spastizin, which interact with AP-5 and canonical fission factors such as clathrin and dynamin, drive putative lysosome-specific fission events [66,67,68]. Large BEACH domain-containing proteins such as LYST1 are recruited to lysosomal membranes and promote appropriate lysosomal fission [69,70]. The lysosomal Ca^2+^ channel TRPML1 is also required for lysosomal fission and fusion, and mutations in *TRPML1* disturb neuronal lysosome trafficking and lead to lysosomal storage disorders (LSDs) [71].

Together, endo-lysosomal sorting and autophagy provide neurons with a constant degradative flux into lysosomal compartments, and lysosomal fission and fusion restore the number and size of lysosomes. Lysosomal degradative capacity directly controls the rate at which cargoes delivered from these two pathways are degraded. The resulting amino acids, short peptides, and other monomeric subunits are exported back into the cytosol via efflux transporters for reuse, and they also serve as signaling molecules to regulate metabolic responses. Thus, degradative flux into lysosomes (from endocytosis and autophagy) and the ability of lysosomes to clear that flux together define neuronal lysosomal capacity—a parameter that appears to change with age (Section 3) and is tuned by TFEB-family signaling (Section 4).

### 2.2. Signaling and Regulatory Roles

Lysosomes act as signaling platforms. The nutrient-sensitive kinase complex mTORC1 is recruited to the cytoplasmic side of lysosomal membranes in response to amino acid availability, where it integrates inputs to promote anabolic processes and inhibit autophagy [4,72,73]. Conversely, AMPK can be activated at lysosomes during energetic stress, promoting catabolic processes and enhancing autophagy [74,75].

Lysosomal compartments participate in intracellular Ca^2+^ signaling. Endolysosomes are a major storage compartment for Ca^2+^: the luminal concentration of free Ca^2+^ is thought to be ~500 μM when the luminal pH is ~4–5, whereas the cytosolic concentration is ~100 nM [76,77,78]. Still, the ER and mitochondria each store more of the cell’s total Ca^2+^ [79]. The lysosomal Ca^2+^ pool is readily releasable through channels including MCOLN1/TRPML1, TPCs, and inositol triphosphate receptor (IP_3_R) [80,81,82]. Indeed, through the IP_3_R, endolysosomal compartments have been shown to release Ca^2+^ in response to IP_3_ in several cell types [81]. Release of Ca^2+^ through endolysosomal IP_3_Rs generates local increases in cytosolic Ca^2+^ concentration, which could be amplified by Ca^2+^ release from the ER through Ca^2+^-gated ryanodine receptor (RYR) Ca^2+^ channels in excitable cells, including neurons [83]. In addition to its involvement in cellular Ca^2+^ signaling, lysosomal Ca^2+^ release influences lysosomal fusion, fission, and trafficking [71,84] and serves as a signal during lysosomal stress by activating calcineurin, promoting TFEB nuclear translocation [85].

In neurons, there are hints that these signaling functions are important for activity-dependent processes. For instance, synaptic activity alters local energy demand and can modulate mTORC1 activity, linking lysosomal signaling to plasticity [86,87]. Whether lysosomal signaling is sufficient to drive specific synaptic plasticity programs in vivo remains under investigation.

Lysosomes also regulate other organelles. For instance, at lysosome-mitochondria contact sites, lysosomes regulate mitochondrial fission [88]. Lysosome-Endoplasmic Reticulum contact sites mediate the exchange of lipids, and their disruption is associated with neurological diseases, including hereditary spastic paraplegia and amyotrophic lateral sclerosis [89].

Furthermore, lysosomes are a major storage site of ions beyond Ca^2+,^ including Fe^2+^, Fe^3+^, Zn^2+^, and Cu^2+^ [90], and it is thought that inter-organellar contact sites mediate transport of ions from lysosomes to other organelles [90,91,92]. Considering the redox-active iron in particular, lysosomes accumulate these cations through degradation of ferritin, heme-containing proteins, and transferrin cargo [80,93]. When this iron is released into the cytosol, such as through lysosomal membrane permeabilization (LMP), it can not only exacerbate cellular damage, including ROS production and lipid peroxidation, but also promote ferroptosis [93,94,95].

The endolysosomal system is involved in intercellular signaling through the secretion of exosomes [96]. Exosomes are the intraluminal vesicles of MVBs that are released into the extracellular space when the MVB fuses with the plasma membrane. Exosomes can regulate neurodevelopment and synaptic function; the field is still in the early stages of understanding where and when exosomes form, what the relevant cargoes are, and the roles of exosomes in intercellular communication [96].

### 2.3. Transport and Spatial Organization

Neuronal morphology poses unique demands for lysosomal positioning and transport. Degradative lysosomes are enriched in the soma, while distal axons and dendrites are sites of high turnover demand [9]. Accommodating this disconnect, endosomes and autophagosomes form in axons and dendrites, and they undergo retrograde transport to fuse with somatic lysosomes [9,97]. Acidified lysosomal compartments containing degradative enzymes are also present in neurites [98,99]. These lysosomal compartments fuse with autophagosomes and endolysosomes, and this fusion promotes their retrograde trafficking by motor proteins along microtubules [7,46]. These lysosomal transport events are not only a mechanism for lysosomal catabolism and signaling but also generate a distinct lysosomal function in that neuronal lysosomes and late endosomes can also act as transport vehicles: lysosomes carry mRNAs along axons and dendrites to sites of local translation, and they also carry ER tubules along neurites [100,101]. Regarding mRNA transport, this coupling may coordinate the anabolic process of local protein synthesis with the catabolic process of cargo degradation.

Together, these features illustrate the complexity of lysosomal organization and function in healthy neurons. An important question, therefore, is how well these processes are maintained over the course of aging.

## 3. Neuronal Lysosomes in Aging

Does the functionality of neuronal lysosomal compartments decline in aging? If so, is neuronal lysosomal dysfunction a driver of neuronal dysfunction with age? Addressing these questions requires a working definition of lysosomal functionality that incorporates the various roles of lysosomes in neurons. We propose a multi-pronged definition: adequate abundance and activity of lysosome-resident proteins, including degradative enzymes and transporters, as well as appropriate lysosome fusion, fission, and trafficking machinery; proper sorting of lysosome-resident proteins to lysosomes; adequate functionality of fission, fusion, and sorting machinery; and intact mechanisms for lysosomal repair and quality control. When a cell’s lysosomal functionality is adequate, lysosomal cargo degradation overall is resolved in a timely manner and without over-reliance on backup mechanisms such as lysosome exocytosis.

Lysosomal dysfunction not only disrupts physiological lysosomal roles but also generates additional cellular stresses. LMP is a central example. When the lysosomal limiting membrane becomes damaged, luminal pH rises, impairing lysosomal degradation and recycling activities. LMP also permits leakage of lysosomal hydrolases and ions, including redox-active iron, into the cytoplasm, which can amplify oxidative stress and trigger cell death [93,102]. LMP may be a critical component for the age-associated accumulation of protein aggregates in the brain, as the lysosomal membrane ruptures enable endocytosed protein aggregates to escape the endolysosomal system and enter the cytoplasm [103]. LMP has been documented in several neurodegenerative contexts, including Parkinson’s disease [104,105] and Niemann–Pick disease [106]. Disease-associated protein aggregates themselves can induce LMP [107,108], suggesting a feedback loop in which lysosomal dysfunction and proteotoxic stress exacerbate each other. In addition, failure to retain luminal Ca^2+^, whether due to LMP or altered flux through lysosomal Ca^2+^ channels such as MCOLN1, perturbs local Ca^2+^ signaling and can further impair lysosomal trafficking and repair responses [109,110].

To evaluate whether neuronal lysosomal functionality declines during aging, we first discuss the methods by which neuronal lysosomal function is assessed. We will highlight challenges to quantifying lysosomal functionality; in particular, many endolysosomal phenotypes, such as increased abundance of early endosomes, could be indicative of a variety of underlying phenomena other than lysosomal dysfunction. Still, by assessing a combination of lysosome-related phenotypes, recent studies have painted a picture of inadequate neuronal lysosomal function in aging. Finally, we address whether neuronal lysosomal dysfunction is a driver of neuronal dysfunction with age, drawing on studies in which lysosomal pathways have been genetically or pharmacologically manipulated.

### 3.1. Approaches to and Challenges of Assessing Lysosomal Functionality

**Figure 2:**
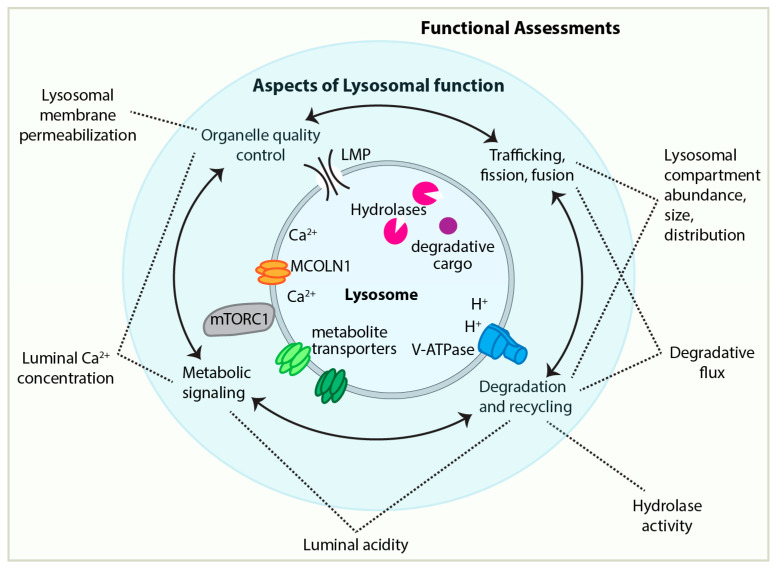
Functional assessment of lysosomal biology encompasses multiple, interrelated aspects of lysosomal function. (Center) Schematic representation of a lysosome. Four major, interconnected lysosomal functionalities are depicted: three that service the cell—degradation and recycling, metabolic signaling, and trafficking—and a fourth, organelle quality control, which is required for the homeostatic maintenance of lysosomal compartment functionality. (Perimeter) Six categories of approaches to assess lysosomal capacity and function.

Here, we briefly consider some of the main approaches used to quantify lysosomal functionality, with emphasis on challenges of interpretation when comparing young versus aging neurons (Figure 2). These approaches, and additional methodologies, have been excellently reviewed in depth elsewhere [6].

Counting lysosomal compartment number and size: With Transmission Electron Microscopy (TEM), lysosomal compartments can be identified based on the single-membrane encapsulation and presence of internal electron dense material and membrane whorls, but the heterogeneity of size and appearance, combined with the presence of other organelles such as autophagosomes that can have a similar appearance, means practice and judgment are necessary [111]. Autophagosomes and endolysosomes can also be identified by their distinctive appearances. Immuno-TEM or correlated light and electron microscopy (CLEM) provides additional information that aids in distinguishing organelles and subpopulations of organelles [112,113,114,115,116]. Volume EM techniques such as serial block-face scanning EM (SBEM) and focused ion beam SEM (FIB-SEM) have increased the throughput of generating 3D EM reconstructions such that sample sizes conducive to quantifying lysosomal compartments are feasible [117,118,119].

For visualizing lysosomal compartments with light microscopy, a challenge is that lysosomal compartments are heterogeneous in size, shape, molecular composition, and pH. Furthermore, lysosomal resident proteins traffic through the secretory pathway at least during biosynthesis. These factors make it challenging to label specific lysosomal compartments. For example, LAMP1, which has been used as a marker for lysosomes, labels not only degradative lysosomes but also late endosomes, autolysosomes, and amphisomes (autophagosomes fused with late endosomes) throughout neurons [120,121,122]. Acidic lysosomal compartments can be visualized using pH-sensitive, live-cell LysoTracker dyes, though the fluorescence intensity cannot be used to measure lysosomal pH [6]. A combinatorial labeling approach helps to identify specific subsets of lysosomal compartments.

Beyond the technical challenges to assessing the number, size and distribution of lysosomal compartments, there is a conceptual challenge for interpreting such data in regard to neuronal lysosomal function, in that the steady-state abundance and localization could mean a variety of things for the cell [123]. For instance, if an aged neuron has increased lysosomal functionality compared to a young neuron, it might have more lysosomes in the soma, enabling faster flux of degradative cargo. On the other hand, if an aged neuron has decreased lysosomal functionality compared to a young neuron, it might likewise have more lysosomes in the soma due to slowed degradation of each load of cargo, leading to a lack of lysosomal fission.

Quantifying lysosomal degradative flux: For cultured neurons, degradative flux can be assessed using fluorescence microscopy by visualizing fluorescent cargoes for lysosomal degradation, typically employed with cargoes internalized through endocytosis. pH-sensitive dyes can help identify when and where these cargoes reach acidified lysosomal lumens. These cargoes can be endocytosed by receptor-mediated endocytosis or bulk endocytosis. For receptor-mediated endocytosis, a challenge is that the dynamics of that specific receptor’s trafficking might change with age in a manner that is specific to the biology of that receptor rather than due to changing endolysosomal flux or lysosomal function. Another consideration is that the rate of endocytosis must be assessed, as that rate will impact the accumulation of degradative flux reporters. Using a combination of both fluorogenic probes, like DQ-BSA, and fluorescent probes, like Texas Red-BSA, distinguishes endocytosis defects and degradation defects [124]. Finally, for dyes that are degraded within lysosomes, the appropriate timing of the experiment is critical so that reduced fluorescence in, for example, aged neurons compared to young neurons can be confidently interpreted as decreased degradative flux rather than increased degradative flux [6].

Degradative flux of a representative lysosomal cargo can be quantified in vivo using genetically encoded tools that incorporate a pulse-chase component, such as Halo tag or the ARGO (Analysis of Red-Green Offset) method [125,126]. However, if turnover is found to be slower in aged animals, additional experiments are required to determine whether the mechanism involves lysosomal dysfunction as opposed to altered regulation of that specific cargo.

Assessing lysosomal acidity: Lysosomal acidity is a good indicator of degradative competence, as most lysosomal hydrolyses operate optimally at low pH [127]. Acidity is also relevant for metabolic signaling because the V-ATPase, which generates the proton gradient across the lysosomal membrane, directly regulates mTORC1 signaling [73].

Relative lysosomal luminal pH can be quantified using ratiometric fluorescence [128]. This can be achieved with dyes such as LysoSensor, which labels all acidic compartments, or with genetically encoded ratiometric reporters in which a lysosomal luminal protein is dually tagged with pH-insensitive mCherry and pH-sensitive GFP [6,21]. Fluid-phase pulse-chase labeling with pH-sensitive and pH-insensitive dyes can also be used, but only compartments actively participating in the endocytic flux will be labeled, limiting the ability to assess total lysosomal capacity [6,129].

Measuring hydrolase activity: In vitro biochemical assays can quantify total lysosomal hydrolase activity in brain lysates [17,20]. These assays provide a general measure of degradative capacity, but they neither distinguish between cell types nor report on subcellular localization. In vitro activity may also overestimate physiological function if lysosomal pH is elevated in situ. Moreover, total hydrolase activity alone does not indicate whether degradative capacity is adequate for cellular needs [17].

In cultured cells, hydrolase activity can be quantified using membrane-permeant fluorogenic substrates [6,130]. Magic Red reagents, for example, contain peptide sequences that confer relative cathepsin selectivity, and fluorescence intensity per cell serves as a proxy for lysosomal function [130].

Lysosome hydrolase activity can also be inferred from the abundance and maturation state of hydrolases. Western blot analysis can quantify total hydrolase protein and the proportion of mature versus pro-enzyme forms [131]. Endoglycosidase H treatment, which degrades proteins without N-linked glycan modifications, can identify immature hydrolases that have not yet trafficked through the Golgi [132]. However, these assays do not report subcellular localization and therefore may miss cases of hydrolase leakage, mis-sorting, or defects in endo-lysosomal fusion.

Hydrolase abundance can be measured in situ using antibodies or fluorescent hydrolase inhibitors such as BODIPY-conjugated probes [6,132,133].

Measuring lysosomal Ca^2+^ concentration: Lysosomes store Ca^2+^, and the leakage of Ca^2+^ from lysosomes into the cytoplasm can be indicative of lysosomal dysfunction [134]. Genetically encoded Ca^2+^ indicators such as GCaMPs are commonly used for measuring Ca^2+^ levels in specific subcellular locations; however, the fluorophore is sensitive to acidic environments [135,136]. Cytosolic GCaMP can be used as an indirect indicator of Ca^2+^ uptake and release from organelles, though whether lysosomes versus other Ca^2+^ storage organelles are involved is unclear. The development of pH-insensitive, bioluminescent, aequorin-based genetically encoded Ca^2+^ indicators has enabled Ca^2+^ measurement of lysosomes [81,135,137]. For cultured cells, another option is chemical Ca^2+^ sensors, such as the fluorogenic calcium-sensitive dye Cal-520, conjugated to dextran so that they will enter the endolysosomal system through endocytosis [78,138].

Lysosomal membrane permeabilization (LMP): LMP can be indicative of lysosomal dysfunction: if the ions and enzymes residing within the lysosome leak into the cytoplasm in an uncontrolled manner, that would both deplete lysosomal functionality and possibly generate toxicity in the cytoplasm.

LMP can be assessed by cytosolic release of 3–10 kDa fluorescent dextran, which works as a marker for endolysosomal flux and cannot be degraded by lysosomes [139]. The accuracy of these measurements requires functional endocytic machinery to load dextran into lysosomes.

Alternatively, LMP can be quantified with Galectin-3/GAL3 recruitment to lysosomes [140,141]. This occurs when the lysosomal resident glycoproteins, which shield the luminal membrane from the low pH and hydrolases, are exposed to the cytoplasmic side, such as when cells are exposed to strong lysosomal membrane-permeabilizing agents [140,141,142]. The physiological circumstances that cause LMP and/or recruit Galectin-3 to lysosomes are still a developing area of research [6]. There are hints that neurodegenerative disease-associated protein aggregates within the lysosomal lumen can cause LMP, described further in Section 3.2.1 [107,143]. Interestingly, neurons exhibit a degree of basal LMP, observed in cultured human induced neurons, human transduced neurons, and mouse pyramidal neurons in vivo [103,138]. Basal LMP is also observed in astrocytes [144]. In light of this, LMP in neurons may not be exclusively indicative of pathology but rather could be a physiologically regulated phenomenon with a yet-unclear cellular function. Indeed, LMP does have physiological roles in other contexts, such as to promote proper chromosome segregation during mitotic cell division [145].

In summary, a neuron’s lysosomal function can be compromised if the lysosomal luminal acidity is not low enough to maintain activity of degradative enzymes, if the abundance of one or more critical degradative enzymes is too low, if Ca^2+^ storage or release is disrupted, or if there is inadequate fusion or fission. Considering this list of disparate phenotypes, there is no one key metric by which lysosome dysfunction can be identified. Instead, assessing lysosomal functionality requires a combinatorial assessment of lysosomal compartment and distribution, lysosomal protein abundance, lysosomal acidity, and lysosomal degradative flux. As an illustration, we consider an example from cultured human fibroblasts: in senescent cells, the average number of Galectin puncta per cell and the average lysosomal luminal pH are both increased compared to non-senescent cells, indicative of lysosomal dysfunction [146]. Senescent cells also have more lysosomal compartments and increased abundance of several lysosomal proteins compared to non-senescent cells, and multiple reporters for lysosomal degradative flux indicated no decrease between senescent versus non-senescent cells [146,147]. Considered together, these phenotypes indicate that senescent cells sufficiently upregulate lysosome abundance to maintain their degradative capacity [146]. Finally, for mammalian neurons, lysosomal function can be most thoroughly assessed in cultured cells, but culture conditions do not fully recapitulate neurons’ morphology, function, and cell-extrinsic environments in the intact nervous system. Confidence in the applicability of in-depth analyses from cultured mammalian neurons is bolstered by similar findings from invertebrate and vertebrate animal neurons in situ [123,148].

### 3.2. Neuronal Lysosomal Dysfunction in Aging: Overview of the Evidence

It has been appreciated for decades that cargoes for neuronal lysosomal degradation accumulate in the brain with age, leading to the hypothesis that neuronal lysosomal function itself declines in aging [17]. Only in the past few years has the question been addressed with the combinatorial methodological approach necessary to generate strong evidence that this is indeed the case.

#### 3.2.1. Hints and Correlations

One lysosomal degradative cargo that accumulates with advanced age is lipofuscin, which is composed mainly of oxidized cross-linked proteins and lipids [149,150]. Lipofuscin largely accumulates within lysosomal compartments, where it is delivered by macroautophagy; it is generally considered poorly degradable, and its accumulation strongly correlates with cellular aging in neurons and other cell types [149,151]. Further, it is postulated to be a cause of lysosomal dysfunction in aging [149,152]. On the other hand, the presence of a subset of non-degradative, non-recycling lysosomal compartments within a neuron does not necessarily imply that the cell’s overall lysosomal capacity is reduced or inadequate—there could still be functional lysosomal compartments working in parallel to the compartments that stably sequester lipofuscin. This notion that a subset of dysfunctional lysosomes could be set aside from a functional lysosomal system within the neuron is speculative.

Another type of cargo for lysosomal degradation that accumulates with age is misfolded protein aggregates, which are cargoes for autophagy [45,153,154]. As these proteins are continuously expressed (as opposed to turned-on in aging), their accumulation could indicate that (1) protein misfolding increases and/or the ubiquitin–proteasome system declines with age, but autophagy is not sufficiently up-regulated and/or not up-regulated at all, (2) autophagic surveillance declines with age such that protein aggregates that would have been identified and degraded in youth no longer are, or (3) autophagic flux is disrupted downstream of the formation of the autophagosome, and neurons and their surroundings accumulate protein aggregates that were ejected or escaped from autophagy. These possibilities are not mutually exclusive. Another aspect of this age-associated accumulation of protein aggregates is that the protein aggregates involved in neurodegenerative diseases, including tau and α-synuclein, propagate between neurons [155]. In this propagation, neurons endocytose protein aggregates [155,156,157]. These aggregates escape the endocytic pathway into the cytosol, where they template misfolding of the neurons’ endogenous proteins, and a major route of escape is from lysosomal compartments through LMP [107,143,158].

A pulse-chase isotope labeling plus mass spectrometry approach to quantify protein half-lives across the proteome in the brains of young adult versus aged mice indicated an overall increase of 20% in protein lifespans in the aged mice [159]. This increase was observed for putative cargoes of both lysosomal and proteasomal degradation, and putative cargoes for lysosomal degradation (identified as such based on their low isoelectric point) were significantly enriched for slowed turnover. This suggests that the flux of lysosomal degradation declines in aging. This could arise from age-associated lysosomal decline in neurons and/or glial cells, though alternate underlying mechanisms such as metabolic adaptations are equally plausible [159].

There is also an abundance of evidence that endosomal, autophagic, and lysosomal compartments change with age in neurons. Aging leads to the accumulation of late endosomes and multivesicular bodies in the presynaptic terminal in mice and *D. melanogaster* neuromuscular junctions (NMJ) [154,160,161]. In aged mouse hippocampal CA1 and PFC, but not CA3, lysosomal compartment size was increased compared to young adults [148]. This could be consistent with increased endocytic flux, decreased lysosomal degradative flux (leading to a backup of endosomal and autophagic compartments), or both. Indeed, neuronal endocytosis is upregulated with aging in cultured neurons [162], and endocytic proteins, including clathrin, dynamin-1, and Rab5, are up-regulated in aged human brains [163]. The correlation between endocytosis and neuron aging has also been noted in *C. elegans*: *C. elegans* can suspend aging while maintaining neuronal function in the dauer state, and in the dauer state, constitutive endocytosis is strongly suppressed [164]. Furthermore, in that context, the suppression of endocytosis contributes to the long-term preservation of dendrite morphology over time, prompting speculation that constitutive endocytosis can promote aging [164].

Whether and how the abundance and functionality of lysosomal proteins change with age in the brain varies between studies. Cathepsin D, the major lysosomal hydrolase, accumulates in the aging rat brain [165,166]. On the other hand, brains from humans without diagnosed neurodegenerative disease exhibited a progressive decline in glucocerebrosidase activity in the substantia nigra with aging [167]. Haploinsufficiency of glucocerebrosidase is one of the most common genetic risk factors of PD, which suggests that the declining glucocerebrosidase activity with age is likely a factor that promotes pathologies of aging rather than an adaptation to aging [168]. In the aging mouse brain, the activity of some lysosomal hydrolases, including beta-glucosidase, is decreased in aged compared to young adults, but the activity of other lysosomal hydrolases is unchanged or increased, complicating the picture [169]. In both the cortex and hippocampus of aged mice (18 months), the level of TFEB protein is reduced compared to younger adults (6 and 12 months), suggestive of decreased lysosomal capacity [170]. Finally, a neuron-specific transcriptional reporter for the activity of HLH-30, the sole *C. elegans* homolog of TFEB, showed decreased activation within *C. elegans* neurons by mid-adulthood, indicative of declining neuronal lysosomal capacity in aging [123,171].

CMA can be considered an aspect of lysosomal function, as the rate-limiting step is LAMP2A-mediated translocation of cargoes across the lysosomal membrane. The abundance of LAMP2A and other CMA proteins in the brain is either unchanged or increased in aged mice compared to young adults [169,172]. Still, the CMA flux in aging may be inadequate: genetic knockdown of LAMP2A pan-neuronally or from excitatory neurons causes accelerated molecular aging phenotypes in the mouse brain, including lipofuscin deposits, ubiquitinated protein inclusions, and other indicators of proteostasis collapse, suggesting that inadequate CMA contributes to the same aging phenotypes in the wild-type genetic background [172,173].

Neurons, with their high metabolic activity and exceptional longevity, are particularly vulnerable to cumulative environmental stress. The term *exposome* refers to the totality of lifetime exposures, including pollutants, pesticides, endocrine disruptors, and dietary metabolites, several of which are linked to oxidative stress and risk for age-related neurodegenerative diseases [174,175]. Chronic oxidative stress can impair neuronal function by oxidizing lipids, proteins and nucleic acids [176,177]. Oxidative stress may directly damage lysosomes through lipid peroxidation or impaired acidification, indirectly disrupt autophagy, or contribute to neuronal dysfunction without overtly altering lysosomal compartments [95,178,179,180,181,182]. Evidence also suggests that reactive oxidative species (ROS) accumulate in aged neurons, and overexpression of extracellular superoxide dismutase (EC-SOD) in aged mice improves learning and memory [183,184,185,186,187]. The “two-hit hypothesis” has been proposed for AD, PD, and several other neurodegenerative disorders, suggesting that both genetic and environmental factors are necessary to trigger these diseases [188,189]. Oxidative stress serves as one of the key environmental factors, is highly associated with the pathology of these neurodegenerative disorders [190,191]. Additional components of the exposome, such as advanced glycation end-products (AGEs), have been detected in Lewy bodies of Parkinson’s disease and in amyloid plaques of Alzheimer’s disease, suggesting involvement in neurodegenerative pathology [192,193,194,195]. Collectively, these findings indicate that environmental exposures can contribute to age-related neuronal disorders, with lysosomal dysfunction representing one potential pathogenic mechanism.

Considering how all these age-associated changes map onto lysosomal function or dysfunction, the important parameter is whether the lysosomal capacity is adequate for the needs of the cell, rather than the total lysosomal capacity per se. Because the need for lysosomal function increases with aging, aged neurons with unchanged or even increased levels of lysosomal proteins compared to young neurons may still have inadequate lysosomal capacity.

#### 3.2.2. Evidence of Neuronal Lysosomal Dysfunction in Aging

Neuronal lysosomal function with aging was assessed in an aging paradigm of cultured primary neurons, wherein neurons at 21 DIV (days in vitro) are considered mature and neurons at 28 DIV are considered aged [148]. Supporting this categorization, 21 DIV neurons had established a maximal number of synapses, and 28 DIV neurons phenocopied several signs of aging in vivo, including accumulation of lipofuscin, but did not show gross morphological changes such as neurite degeneration [148]. Notably, the aged neurons showed decreased lysosomal compartment acidity compared to mature neurons [149]. Furthermore, the DQ-BSA intensity within lysosomal compartments throughout the neurites, but increased intensity of Dextran-647. As Dextran-647 fluoresces constitutively but DQ-BSA only fluoresces upon proteolytic cleavage within lysosomal compartments, these data show that bulk endocytosis is increased, but degradative flux is decreased, in the aging cultured neurons [148]. In addition, compared to the mature neurons, the aged neurons showed a redistribution of lysosomal material. Specifically, the aged neurons had increased size and number of lysosomal compartments labeled by LAMP1 and increased ratio of cathepsin D to LAMP1 in lysosomal compartments containing both, but no obvious change in the total abundance of LAMP1, total cathepsin D, or mature (protease-cleaved) cathepsin D.

The question of how aging impacts neuronal lysosomal function was also recently addressed using cultured human neurons that were transdifferentiated from fibroblasts of young adults (~25 years old) versus older adults (~70 years old) [138]. Compared to those from young adults, tNeurons from older adults showed decreased abundance of lysosomal proteins by proteomic analysis, decreased lysosomal Ca^2+^, decreased Cathepsin B activity, and no detectable change in lysosomal acidity. They also showed increased basal levels of LMP as well as slower kinetics of repairing LMP induced by exposure to the lysosomotropic drug LLOME (L-leucyl-L-leucine *O*-methyl ester). Altogether, these results paint a picture of decreased neuronal lysosomal capacity and function in aging [138].

CMA flux within neurons, assessed by genetically expressing the CMA-targeting motif KFERQ tagged to the fluorescent protein Dendra, tended to be reduced within the neurons of aged mouse brain compared to young adults [196]. Indeed, most organs and cell types showed reduced CMA flux in aged mice. The changes were dependent on neuron type and animal sex, though: C1 and DG hippocampal neurons and Purkinje neurons of both males and females showed reduced CMA flux in aged adults, entorhinal cortex neurons showed increased CMA flux in older animals, and somatosensory cortex neurons showed decreased CMA flux in aged males but not females [196].

In *C. elegans*, neuronal lysosomal compartments show increased luminal pH with aging [123]. They also exhibit an increased proportion of lysosomes with an abnormally high accumulation of a representative cargo for lysosomal degradation, the transmembrane synaptic vesicle protein Synaptotagmin/SNT-1, and slowed turnover of another transmembrane synaptic vesicle protein, Synaptogyrin/SNG-1 [123]. Unlike lipofuscin, Abeta, and similar, these lysosomal degradative cargoes are unlikely to be damaged such that they are challenging to degrade; therefore, this alteration in their degradative flux more directly implicates lysosomal dysfunction [123].

Considering these studies together, the interpretation of age-associated lysosomal dysfunction is bolstered.

#### 3.2.3. Functional Consequences of Neuronal Lysosomal Dysfunction

Does it matter for neuronal health when the lysosomal capacity is inadequate, and if so, in what regard? As described above, lysosomes perform a variety of functions in the neuron; therefore, neuronal lysosomal dysfunction is expected to impact the neuron in multiple aspects. Experiments in which neuronal lysosomal function is genetically or chemically manipulated to assess the outcome on neuron maintenance have shed light on this question. There is a wealth of experimental evidence showing that causing lysosomal dysfunction accelerates neuronal aging phenotypes and functional decline; this indicates that lysosomal function is necessary to prevent aging phenotypes. The most compelling evidence also includes showing that preserving lysosomal function into aging delays age-related neuron dysfunction, as this suggests that lysosomal functionality is limiting for neuronal maintenance. Here, we highlight studies that incorporate that latter genre of evidence.

The accumulation of protein aggregates with age is perhaps the most proximal pathology to arise from age-associated decline in neuronal lysosomal function. In human tNeurons from aged individuals, treatment with compounds that improve lysosomal function led to decreased levels of Aβ42, the accumulation of which contributes to AD, whereas treatment with compounds that exacerbate lysosomal dysfunction led to increased accumulation of Aβ42 [138]. Aged, cultured neurons show reduced synapse numbers compared to younger neurons; pharmacologically restoring lysosomal acidity in the aged, cultured neurons partially restored synapse numbers, while pharmacologically reducing lysosomal acidity caused the decrease in synapse number to occur earlier [148]. These studies from cultured neurons indicate that neuronal lysosomal function is instructive for preventing both pathological protein buildup and declining neurotransmission in aging.

Several studies from intact animals corroborate the key role of neuronal lysosomal function in maintaining neuronal function in aging. Over-expression of TFEB in neurons, which is expected to increase lysosomal capacity and function, caused improved learning and memory in old mice [170]. In *D. melanogaster*, neuronal over-expression of the CMA effector LAMP2A bolstered climbing performance during aging without extending lifespan [197]. In *C. elegans*, the impaired neuronal lysosomal acidification that occurs during aging drives structural deterioration of dendrites [123]. Loss of TFEB/HLH-30 likewise accelerated age-associated dendrite morphological defects, whereas over-expression of TFEB/HLH-30 improved them [123].

For the cargo degradation function of lysosomes, it is worth considering that lysosomal degradation need not be resolved cell-intrinsically. Neuronal lysosomal degradative cargoes can be released to the extracellular space through several mechanisms. Lysosomal exocytosis, originally thought to be a specialized function of hematopoietic cells, has been identified in an increasingly wide variety of mammalian cell types [198,199], and it provides a cell-non-autonomous route for removal of cellular garbage [200,201]. A second mechanism is MVB fusion with the plasma membrane, releasing exosomes [96]. Exosome release can serve as a backup for lysosomal dysfunction in that disruption of lysosomal acidity causes increased exosome release [202,203,204]. A third mechanism is through the release of ectosomes, which contain cytosolic material that buds out from the plasma membrane [96]. For instance, in *C. elegans*, would-be cargoes for lysosomal degradation, including protein aggregates and organelles, can be secreted from neurons as large vesicles called exophers [205]. Neuronal exopher secretion increases upon inhibition of many canonical autophagy genes, and surprisingly, this generates a lifespan extension [206]. Therefore, this is a case where reduced neuronal autophagic flux is beneficial, rather than harmful, for neuronal health [206].

Mammalian neurons employ these backup mechanisms for inadequate lysosomal degradation by secreting pathogenic protein aggregates involved in neurodegenerative diseases. For example, neuron-expressed human α-synuclein is degraded by microglia in the mouse brain, which prevents neurodegeneration [207]. In fact, a few hundred aggregation-prone neuronal proteins can be found within microglia in the aging mouse brain, indicative of widespread neuronal outsourcing of protein degradation [208]. Unfortunately, the outsourcing of neuronal lysosomal degradation, combined with inadequate lysosomal degradation in the cells that uptake the waste, is a mechanism by which pathogenic protein aggregates spread and propagate between neurons, leading to neurodegenerative disease [23,209]. Furthermore, though the degradative functions of neuronal lysosomes can be outsourced to other cells, neuronal lysosomes provide anabolic building blocks and participate in trafficking functions, and outsourcing the degradation of intracellular waste leaves these challenges to resolve.

Another mode by which neuronal lysosomal dysfunction promotes functional decline in the brain is through inflammation. Aged tNeurons generated from healthy individuals as well as those generated from individuals with sporadic AD are more prone to inflammasome activation, and both secrete more pro-inflammatory cytokines, compared to tNeurons from young adults [138]. Connecting that inflammation to lysosomal dysfunction, cytokine secretion can be induced from young adult tNeurons by incubation with a drug that disrupts the lysosomal membrane, LLOME, and cytokine secretion can be ameliorated in the sporadic AD neurons by incubation with a drug that promotes lysosomal function, C381 [138]. These inflammatory signals can cause toxicity to neurons, microglia, and astrocytes [210]. In addition, impaired autophagy can lead to activation of the cyclic GMP-AMP (cGAMP) synthase (cGAS)/stimulator of interferon genes (STING) pathway, which drives inflammation in microglia and neurodegeneration in aging [211,212].

Finally, lysosomal dysfunction also causes or contributes to several neurodegenerative disorders. Mutations that impair lysosomal homeostasis lead to abnormal accumulation of macromolecules and result in LSDs [213]. In the nervous system, these defects promote axonal degeneration, inflammation and neuronal death, and more than half of LSDs present with psychiatric or cognitive symptoms [214,215,216,217]. For instance, a mouse model of Niemann–Pick type C (NPC) disease exhibits transcriptional and functional features consistent with accelerated brain aging [218]. Further, NPC mouse tissue shows transcriptomic similarities to human AD samples [218]. Both aging and AD neurons exhibit constitutive lysosomal damage and impaired lysosome repair, and restoring autophagy or lysosomal function mitigates AD-related defects such as Aβ accumulation and inflammation [139,219,220,221,222]. Lysosomal and autophagic-related abnormalities are also present in PD, where mutations in several common risk genes, including *LRRK2*, *ATP6AP2*, and *SNCA*, have been implicated in impaired lysosomal acidification and abnormal accumulation of undegraded cargo within lysosomes [223,224,225]. In both AD and PD, lysosomal dysfunction co-occurs with age-related hallmarks such as lipofuscin accumulation, oxidative stress, and inflammation [192,226,227,228,229]. Altogether, lysosomal dysfunction not only contributes to neurodegenerative pathology but can also manifest as features resembling accelerated neuronal aging.

#### 3.2.4. Autophagic Dysfunction, Upstream of Lysosomal Function, with Age

There are many mechanisms of neuronal aging that likely function in parallel, at least partially, to lysosomal function [230]. By contrast, macroautophagy and lysosomal function are partially overlapping Venn diagrams. Therefore, we consider here age-associated dysfunction of autophagy upstream of lysosomal function, since the two are so connected. Specifically, there is evidence that insufficient autophagosome formation, as opposed to insufficient lysosomal functionality, is a driver of neuronal pathologies of aging [231].

Macroautophagy becomes dysfunctional in aging neurons in *C. elegans*, *D. melanogaster*, and mice [232,233,234,235,236,237,238,239,240]. For mice, work in cultured Dorsal Root Ganglion neurons isolated from adult mice at different ages, combined with characterizations of the aging neuromuscular junction in vivo, showed that the rate of autophagosome formation in distal neurites progressively declines with age, with no obvious defect in acidification nor retrograde transport of autophagosomes in aging [234,235]. Likewise, autophagic flux was reduced in hypothalamic proopiomelanocortin (POMC) neurons in the aged mouse brain [241]. In *D. melanogaster*, *Atg8a* loss-of-function causes shortened lifespan and more rapid onset of accumulation of insoluble ubiquitinated proteins, and over-expression of *Atg8a* in the brain causes a dramatic lifespan extension and reduced accumulation of damaged macromolecules—would-be cargoes for lysosomal degradation—in the brain [242]. ATG8 is involved in autophagosome formation and fusion with lysosomes, and so it is upstream of lysosomal function [243]. Along similar lines, loss-of-function of ER-phagy (selective autophagy of the Endoplasmic Reticulum) receptors leads to neurodegeneration, and neuron-specific up-regulation of the ER-phagy receptors extends lifespan and enhances motor function in aged *D. melanogaster* [244]. Neuronal over-expression of BNIP3, a mitochondrial protein and inducer of autophagy, was sufficient to partially ameliorate age-associated decline in neuronal mitophagy in *D. melanogaster* [233]. In *C. elegans*, activation of neuronal autophagy delays age-associated neurodegeneration and extends lifespan [232,245]. Of note, in *D. melanogaster*, genetic hyperactivation of autophagy in aging non-cell-autonomously antagonizes age-associated decline in neuron function and extends lifespan [233,246]. That non-cell-autonomous signaling role is likely a parallel mechanism to lysosomal function/dysfunction.

In summary, aging neurons exhibit multiple, partially overlapping failures in maintenance relating to lysosomal degradation, from reduced autophagosome formation to impaired lysosomal acidification. Together, these changes lower the neuron’s effective lysosomal capacity and constrain its ability to recover under stress. Given these age-associated deficits in lysosomal function, a central question is how neurons set and preserve lysosomal capacity across adulthood. We therefore next discuss TFEB and allied pathways that scale lysosomal capacity, as well as mechanisms of lysosomal quality control that preserve lysosomal function.

## 4. Regulation of Lysosomal Functional Capacity: TFEB and Quality Control

An understanding of how neuronal lysosomal capacity is regulated in healthy, young neurons is useful for assessing and counteracting age-associated neuronal lysosomal dysfunction. Lysosomal biogenesis and renewal rely on the coordination between gene expression, protein synthesis, and secretory pathway dynamics [1]. The Endoplasmic Reticulum (ER), Golgi apparatus and endosomes ensure the delivery of newly synthesized membrane and luminal proteins, including hydrolases, ion channels and transporters, into lysosomes [2]. Generating a cell’s lysosomal capacity involves transcription of lysosome and lysosome-related genes, and protein production and trafficking. It also requires quality control mechanisms to detect and resolve lysosomal stress—circumstances or defects that impair lysosomal function.

### 4.1. TFEB-Mediated Lysosomal Biogenesis

**Figure 3:**
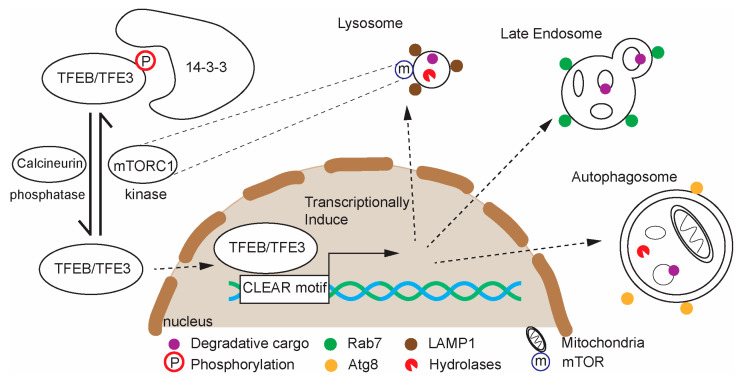
Schematic of TFEB and TFE3 regulation and function in lysosomal gene expression. TFEB and its homologs regulate genes involved in lysosome, endolysosome, and autophagosome biogenesis. TFEB is most prominently regulated by mTORC1, which phosphorylates TFEB, promoting its cytoplasmic retention.

TFEB promotes transcription of many genes that encode lysosomal and autophagic proteins as a suite, and it is therefore often called a “master regulator” of lysosome biogenesis [247,248] (Figure 3). TFEB activity is regulated by phosphorylation: under nutrient-rich conditions, mTORC1 phosphorylates TFEB, causing its cytoplasmic retention [249,250,251]. During starvation, stress, or lysosomal dysfunction, TFEB is dephosphorylated, allowing it to translocate to the nucleus and activate gene expression [247,252]. TFEB function is regulated by several other signaling mechanisms beyond mTORC1. These include negative regulation from extracellular signal-regulated kinase 2 (ERK2), positive regulation by the phosphatase calcineurin when Ca^2+^ leaks from the lysosomes during lysosomal stress, positive regulation by AMPK, and positive regulation by protein phosphatase 2A (PP2A) [247]. As an integrator of these signaling pathways, TFEB sits at the intersection of nutrient sensing and lysosomal homeostasis. Furthermore, TFEB can be activated by lysosomal damage/stress, which can include the release of Ca^2+^ from the lysosome, and it is therefore part of a lysosome quality-control response [134]. In mammals, Transcription Factor E3 (TFE3) and MITF, members of the Mit/TFE family of transcription factors, along with TFEB, play some partially overlapping roles as TFEB in lysosomal biogenesis [253]. Despite their partial functional redundancy, the presence of both TFEB and TFE3 is required for maximal lysosomal biogenesis and autophagy in response to starvation or stress [254].

TFEB has garnered attention as a modulator of neurodegenerative disease progression: overexpression of TFEB can improve cellular and organismal health in several murine neurodegenerative disease models, including models of Parkinson’s, Huntington’s and Alzheimer’s disease, by inducing autophagy to degrade toxic protein aggregates, and depletion of TFEB can cause accelerated disease progression [87,247,255,256,257].

TFEB also promotes neuronal autophagy, including lysosomal biogenesis, in non-diseased neurons. In *D. melanogaster*, both brain-specific over-expression of TFEB/Mitf and brain-specific knockout cause increased abundance of autophagosomes [258]. A plausible interpretation of these data is that TFEB/Mitf sets the rate of autophagic flux: excessive Mitf leads to increased autophagosome formation, while lack of Mitf causes autophagosomes to accumulate due to diminished lysosomal capacity [258]. In *C. elegans*, TFEB/HLH-30 appears to be dispensable during neurodevelopment but expands neuronal lysosomal capacity in early adulthood [124]. Furthermore, neuronal TFEB may play a role in regulating synaptic communication in some contexts: in cultured rat primary cortical neurons, chronic suppression of synaptic inactivity activates TFEB, which promotes homeostatic synaptic up-scaling [86].

In addition to TFEB and TFE3, several other transcription factors, transcriptional repressors, and epigenetic regulators contribute to the control of lysosomal biogenesis. In mouse cell culture, deficiency in lysosomal cysteine proteases or increased endocytic substrate load leads to elevated lysosomal oxidative stress, which activates the transcription factor STAT3 and induces expression of lysosomal proteolytic hydrolases [259]. MYC, an E-box-binding transcription factor, acts as a transcriptional repressor of lysosomal biogenesis by occupying promoters of lysosomal genes and antagonizing TFEB/TFE3 activity; overexpression of MYC suppresses lysosomal and autophagic function in pluripotent stem cells and cancer cells [260]. Glucose starvation produces genome-wide demethylation of H3 Arg17, inducing nuclear translocation of the arginine methyltransferase CARM1, which cooperates with TFEB to serve as a transcriptional coactivator of lysosomal and autophagosomal biogenesis [261]. However, to date, there is no direct evidence regarding whether these regulatory mechanisms influence lysosomal biogenesis in neurons.

### 4.2. Lysosomal Quality Control

The integrity of the lysosomal limiting membrane is continuously under stress: as degradative substrates are delivered, processed, and exported, the osmotic composition of the lumen shifts dynamically, producing fluctuations in membrane tension and lysosomal volume [143]. The high acidity, hydrolytic enzymes, and relatively high concentrations of cations inside the lysosomal lumen also present a peril to the membrane. To withstand these stresses, lysosomal compartments rely on protective mechanisms that broadly fall into four categories: (1) intrinsic membrane-protective components; (2) mechanisms that sense and repair lysosomal damage; (3) mechanisms that expand lysosomal capacity through transcriptional regulation (TFEB and homologs from Section 4.1); and (4) pathways that remove damaged lysosomes. The inducible quality control mechanisms in categories 2–4 can occur simultaneously, and multiple repair pathways within category 2 function in parallel using distinct molecular machinery [262]. We briefly overview lysosomal quality control mechanisms here; several in-depth reviews have been published recently [143,262,263].

(1) Intrinsic protection: The first line of defense against these stresses is intrinsic components of the lysosomal compartments, including the integral membrane glycoproteins, which protect the luminal membrane from damage, and ion channels and transporters [142,264,265].
(2) Sense and repair damage: LMP, Ca^2+^ efflux, and/or disrupted lysosomal acidity can activate several partially overlapping repair pathways. Ca^2+^ efflux from the lysosomal lumen is both a hazard and a signal: elevation of local cytosolic Ca^2+^ rapidly recruits ESCRT machinery to the lysosomal membrane, which promotes repair [266,267,268,269]. Annexin A7 promotes lysosome repair in parallel to ESCRT-III [270]. The mechanisms of both ESCRT- and Annexin A7-mediated repair are not fully understood, but both are thought to involve inducing negative membrane curvature from the cytosolic face of the lysosomal membrane [266,267,268,269,270]. Stress granules form at sites of LMP, stabilizing the membrane and facilitating repair through both ESCRT-dependent and independent mechanisms [271]. LMP can also be repaired by the phosphoinositide-initiated tethering and lipid transport (PITT) pathway, which involves transfer of phosphatidylserine and cholesterol from the ER to the lysosomal membrane [272,273]. Another way in which the ER participates in repairing damaged lysosomes uses the ER-resident, lipid-transport protein VPS13C; the cytosolic side of VPS13C associates with damaged lysosomal membrane, likely to facilitate the transport of lipids from the ER to the lysosome [274].
  The conjugation of ATG8 to single membranes (CASM) pathway provides another major repair mechanism. CASM is activated by lysosomal deacidification or ionic imbalance to repair the lysosome [25,275]. Activation of CASM at lysosomal compartments can occur through two parallel mechanisms: one involves flipping sphingomyelin from the lysosome luminal to cytosolic side [276], and the other involves V-ATPase recruitment of ATG16L1 to the lysosome [25,277].
(3) Stress-responsive expansion of lysosomal capacity: Efflux of Ca^2+^ from the lysosomal lumen can activate calcineurin, which dephosphorylates TFEB to promote transcriptional expansion of lysosomal and autophagic capacity [85]. The CASM machinery also facilitates activation of TFEB/TFE3 during lysosomal stress [278].
(4) Removal of damaged lysosomes: When repair mechanisms are insufficient, damaged lysosomes can be selectively eliminated by lysophagy—selective autophagy of the lysosome [266]. To initiate lysophagy, LMP is sensed by cytosolic galectins, which bind lysosomal luminal beta-galactosides that become exposed by the membrane rupture [279,280,281,282]. Some of these galectins induce ubiquitination of the lysosome, triggering lysophagy through a mechanism that involves recruitment of VCP/p97 [108,280,282,283]. Pathogenic mutations in VCP/p97 cause disorders including frontotemporal dementia, amyotrophic lateral sclerosis, and Parkinsonism; in addition to promoting lysophagy, VCP/p97 is also an effector of macroautophagy, and both of these functions likely contribute to neuron pathologies [284,285]. Damaged lysosomes can also be removed from the cell via lysosomal exocytosis [286].

Beyond activating lysosome quality control pathways, lysosomal stress also interfaces directly with metabolic signaling. Lysosomal stress granules formed at sites of LMP repress mTORC1 activity in several cell types, including neurons [287]. In addition, galectins recruited to damaged lysosomes negatively regulate mTORC1 and activate AMPK, which not only activates TFEB to expand lysosomal capacity but also links lysosomal stress to broader shifts in cellular metabolic state [288].

## 5. Outlook

The body of evidence suggests that neuronal lysosomal function declines with age, and this decline contributes to neuronal dysfunction. The mechanisms that drive this decline remain incompletely defined. Understanding them is important for designing strategies to preserve lysosomal capacity, thereby extending neuronal health-span. A key challenge is to determine what limits a neuron’s ability to sense and repair lysosomal dysfunction to maintain lysosomal capacity [212].

TFEB is one key player that is both necessary and rate-limiting for sustaining neuronal lysosomal capacity [123,170,258]. How TFEB activity is tuned in the context of neuronal aging remains unclear. In principle, TFEB should respond to lysosomal stress, but current evidence indicates that age-associated lysosomal dysfunction does not robustly activate it. Indeed, neuronal HLH-30/TFEB activity appears to decline during aging despite lysosomal impairment [123]. Identifying molecular checkpoints that prevent TFEB reactivation in aging neurons could reveal new therapeutic opportunities.

Lysosomal quality control mechanisms, some of which involve TFEB activation, sustain lysosomal capacity in various cellular contexts. The extent to which these pathways function, adapt, or fail in neurons during adult maintenance is still poorly understood.

Altogether, defining how lysosomal quality-control, metabolic signaling, and transcriptional feedback interact during neuron aging will be crucial for understanding what causes lysosomal capacity to decline and how it might be restored.

## Data Availability

No new data were created or analyzed in this study.

## Abbreviations

The following abbreviations are used in this manuscript:

AMPK	AMP-activated protein kinase
mTORC1	Mechanistic target of rapamycin complex 1
AD	Alzheimer’s disease
PD	Parkinson’s disease
ILV	Intraluminal vesicle
RAB5	Ras-related protein in brain 5
RAB7	Ras-related protein in brain 7
VAMP7	Vesicle-associated membrane protein 7
BORC	BLOC-1-Related Complex
HOPS	Homotypic fusion and protein sorting
LYST	Lysosomal trafficking regulator
TFEB	Transcription factor EB
TFE3	Transcription factor E3
CMA	Chaperone-mediated autophagy
BEACH	Beige and Chediak-Higashi domain
LSD	Lysosomal storage disorder
MCOLN1	Mucolipin-1
TRPML1	Transient receptor potential mucolipin 1
IP3	Inositol Trisphosphate
IP3R	Inositol triphosphate receptor
RYR	Ryanodine receptor
MVB	Multivesicular body
TEM	Transmission electron microscopy
CLEM	Correlated light and electron microscopy
SBEM	Serial block-face electron microscopy
FIB-SEM	Focused ion beam scanning electron microscopy
LAMP1	Lysosomal-associated membrane protein 1
BSA	Bovine serum albumin
ARGO	Analysis of red-green offset
GFP	Green fluorescent protein
GCaMP	Genetically encoded calcium indicator
LMP	Lysosomal membrane permeabilization
NMJ	Neuromuscular junction
CA	Cornu ammonis
PFC	Prefrontal cortex
LAMP2A	Lysosomal-associated membrane protein 2A
ROS	Reactive oxidative species
EC-SOD	Extracellular superoxide dismutase
AGE	Advanced glycation end-products
DIV	Days in vitro
LLOME	L-leucyl-L-leucine O-methyl ester
SNT	Synaptotagmin
SNG	Synaptogyrin
Aβ	Beta-amyloid
STING	Stimulator of interferon genes
NPC	Niemann–Pick type C
POMC	Proopiomelanocortin
ATG8	Autophagy-related protein 8
ER	Endoplasmic reticulum
ERK2	Extracellular signal-regulated kinase 2
PP2A	Protein phosphatase 2A
STAT3	Signal transducer and activator of transcription 3
MYC	Myelocytomatosis oncogene
CARM1	Coactivator-associated arginine methyltransferase 1
BNIP3	BCL2/adenovirus E1B 19 kDa protein-interacting protein 3
ESCRT	Endosomal sorting complexes required for transport
PITT	Phosphoinositide-initiated tethering and lipid transport
CASM	Conjugation of ATG8 to single membranes
